# First person – Nicola Graham

**DOI:** 10.1242/bio.062758

**Published:** 2026-06-26

## Abstract

First Person is a series of interviews with the first authors of a selection of papers published in Biology Open, helping researchers promote themselves alongside their papers. Nicola Graham is first author on ‘ Conserved roles of GATA4 and its target gene *TBX2* in regulation of human cardiogenesis’, published in BiO. Nicola conducted the research described in this article while a PhD student in Dr Branko Latinkic’s lab at Cardiff University, Cardiff, UK. She is now a Senior Scientist in the lab of Dr Michael Graz at Biophys Ltd., Cardiff, UK, investigating the genetic control of cardiac development.

**Figure:**
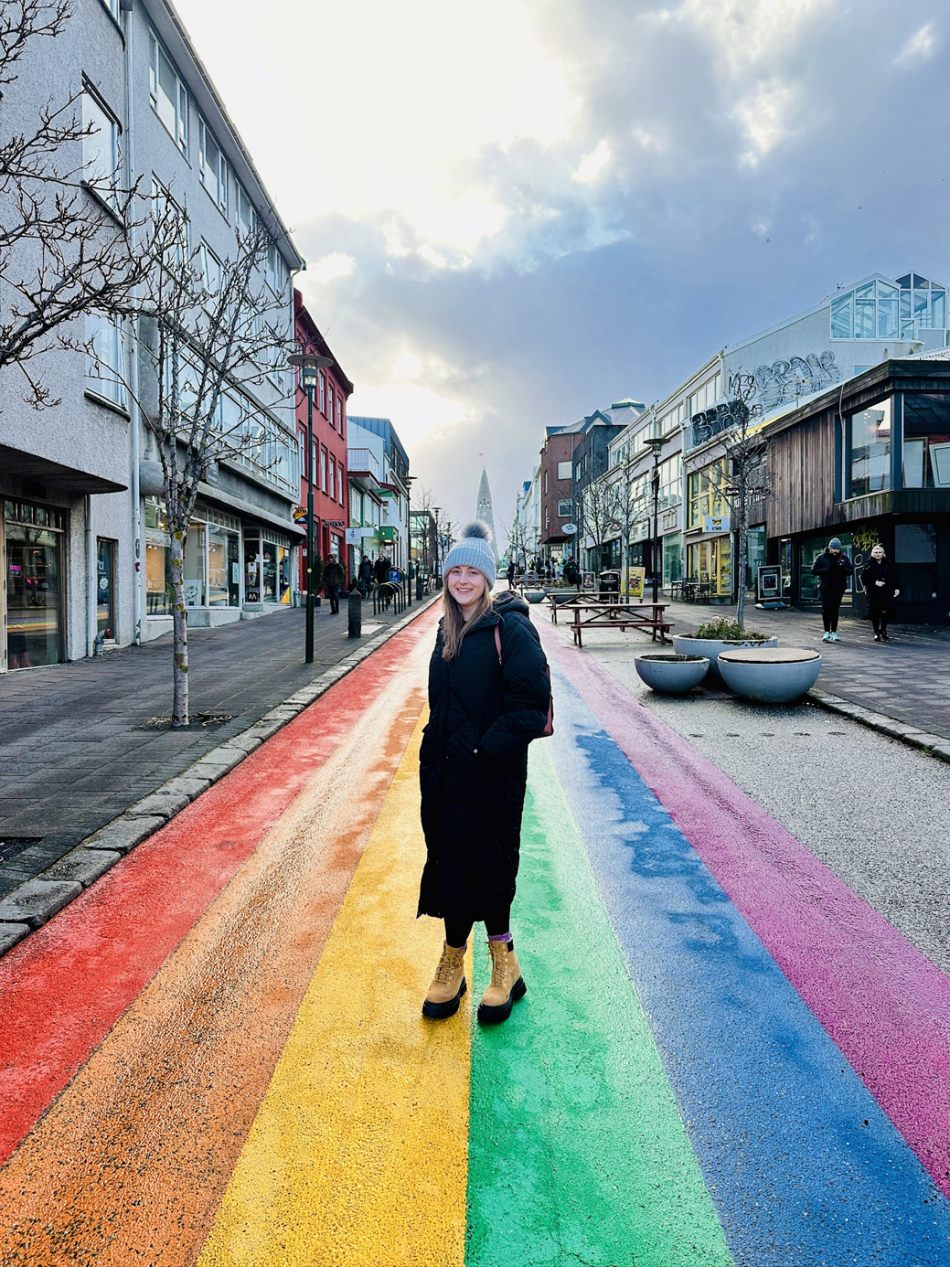
Nicola Graham

**Describe your scientific journey and your current research focus**

My journey into science began much like many others will have, with brilliant teachers who made the subject enjoyable. This inspired me to study biology as a degree at Cardiff University, where I found a passion for developmental biology. Through my course I had the opportunity to undertake a professional training year in Dr Branko Latinkic’s lab, working on the project that would eventually evolve into my PhD project and the paper, ‘Conserved roles of GATA4 and its target gene *TBX2* in regulation of human cardiogenesis’.

Currently, I'm working for BioPhys Ltd., a biotech consultancy based in Cardiff. Our team has a wide breadth of expertise covering pharmaceutical development, healthcare devices, diagnostics and microbiology. I was brought on to expand the business' capacity for projects requiring mammalian cell culture expertise, but working at BioPhys Ltd. has allowed me to engage with a diverse array of projects. Gaining this exposure is exactly what a career in science should be about, continually learning and engaging with new ideas and projects.

Despite moving into an industry role, I still retain a strong passion for developmental biology, particularly cardiomyocte development. They are undeniably some of the most interesting and pretty cells to work with – in my completely unbiased opinion.

**Who or what inspired you to become a scientist?**

As above, the inspiration to become a scientist stems from the influence of great teachers and the desire to have a challenging career that hopefully contributes something positive to the world.

**How would you explain the main finding of your paper?**

Broadly, the cells in our body contain the same genetic blueprint, yet the cells in our heart perform very different functions than those that make up our skin, for example. This is because, in a given cell, only a subsection of genes are ‘turned on’. The genes that are on or off in a given cell decide its fate and its function. Whether a gene is on or not is in part decided by genetic switches like GATA4, which is the focus of the paper.

The paper builds on extensive work in animal models that established GATA4 as a key factor in fating cells to become cardiomyocytes, the workhorse cells of the heart. One of our main findings in the paper is that cardiomyocytes struggle to establish and maintain their identity when GATA4 is absent, and this difference was found to be more dramatic than previously seen in animal models. This work highlights the need for further investigation of GATA4 in human models of heart development to unpick these differences and gain a clearer understanding of the genetics underlying human heart development and disease.

One of our main findings in the paper is that cardiomyocytes struggle to establish and maintain their identity when GATA4 is absent

**Figure:**
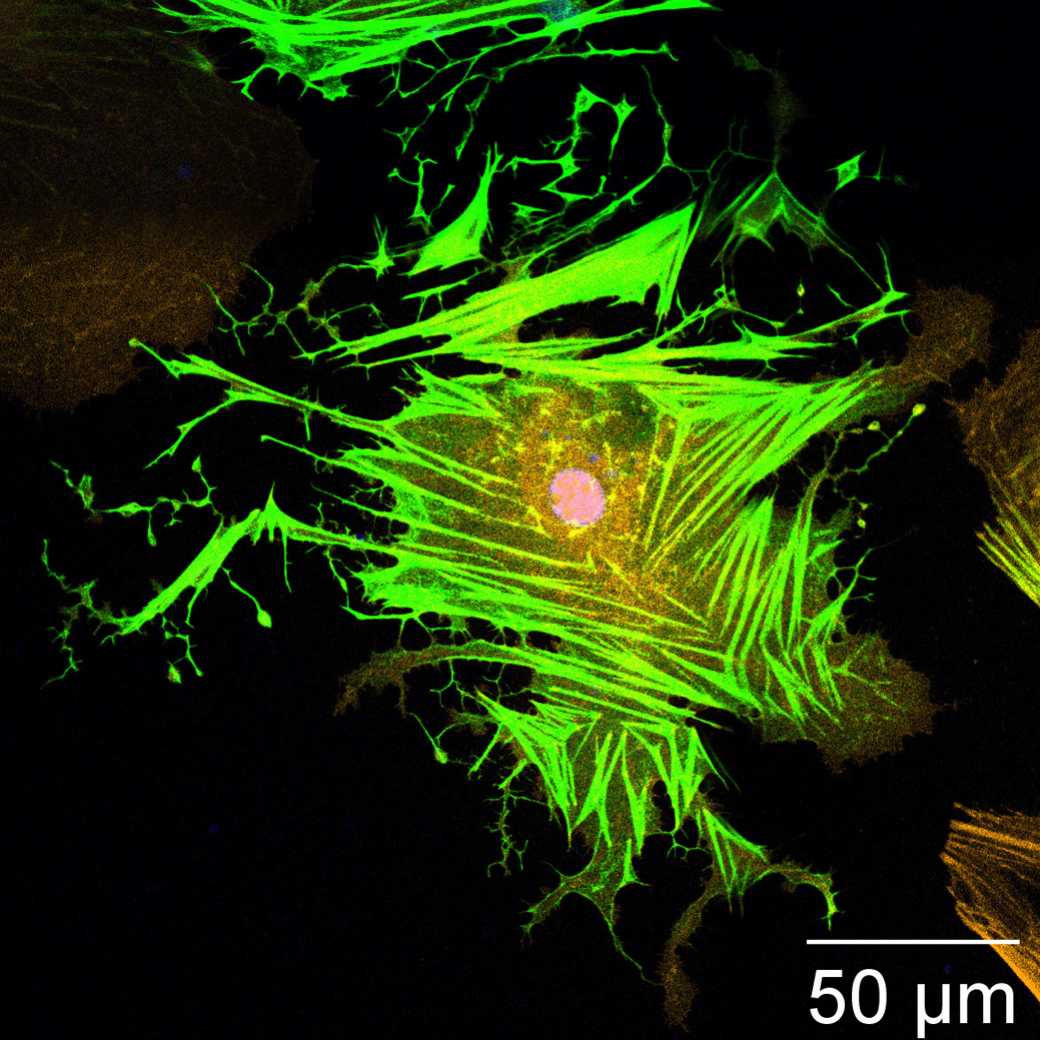
**Am I having a cellular identity crisis?** An immunofluorescent image showing a GATA4-null cell at day 32 of differentiation, 40× magnification. The cell is stained for TNNT2 (green) and Cell Mask Orange (orange), and with DAPI (blue) for nuclear staining. TNNT2 is a specific marker of cardiomyocytes; however, the long-convoluted branching structure of the cell is reminiscent of a myofibroblast, a cell type that is typically TNNT2 negative.

**What are the potential implications of this finding for your field of research?**

There was an observed increase in the expression of fibroblastic genes in the GATA4-null cells, which could be attributed to a few distinct mechanisms. It may reflect a wholesale fate switch, resulting in a higher proportion of fibroblasts compared to the wild type. Alternatively, it could indicate that fibroblastic gene expression is globally derepressed, or perhaps a combination of both. Given the profound impact fibrosis has on cardiac disease progression, these findings strongly warrant further investigation.

**Which part of this research project was the most rewarding?**

This paper features data and cell lines generated during my PhD project. True to the classic PhD experience, some of these data came together right in the nick of time! Successfully generating the GATA4-null line described in the paper was probably the most rewarding, and getting such a strong phenotype to describe. Seeing the final product in print is also very satisfying!

… science is built on lots of people doing things that didn't work until we eventually get to the ‘right’ things

**What piece of advice would you give to the next generation of researchers?**

I actually have three pieces of advice! (1) If you have the opportunity, undertaking a placement training year is a great way of getting the experience you need to decide whether you enjoy working in the lab. An important aspect of this is to make sure you are joining a lab where you can get stuck into lots of different techniques. (2) During the times when absolutely nothing seems to work, remember that science is built on lots of people doing things that didn't work until we eventually get to the ‘right’ things. This sometimes requires delusional levels of faith that it will all work out in the end. (3) When experiments aren't going as planned, breaks and seeking support is essential. Old problems need fresh eyes!

**What's next for you?**

I'm sure there's plenty more in store. You will forever be busy in science, but I feel incredibly fortunate to have been able to follow my interests and build a career out of them. I hope to continue on this path and look forwards to contributing further to the GATA4 story in the future.
